# Seamless recording of glucometer measurements among older experienced diabetic patients – A study of perception and usability

**DOI:** 10.1371/journal.pone.0197455

**Published:** 2018-05-25

**Authors:** Peter Rasche, Alexander Mertens, Talya Miron-Shatz, Corinne Berzon, Christopher M. Schlick, Michael Jahn, Stefan Becker

**Affiliations:** 1 Institute of Industrial Engineering and Ergonomics of RWTH Aachen University, Aachen, Germany; 2 Center for Medical Decision Making, Business School, Ono Academic College, Kiryat Ono, Israel; 3 Center for Medicine in the Public Interest, New York, NY, United States of America; 4 Department of Nephrology, University Duisburg-Essen, Essen, Germany; Medical University of Vienna, AUSTRIA

## Abstract

Self-measurement and documentation of blood-glucose are critical elements of diabetes management, particularly in regimes including insulin. In this study, we analyze the usability of iBG-STAR, the first blood glucose meter connectable to a smartphone. This technology records glucometer measurements, removing the burden of documentation from diabetic patients. This study assesses the potential for implementation of iBG-STAR in routine care. Twelve long-term diabetic patients (4 males; median age of 66.5 years) were enrolled in the study. N = 4/12 reported diabetic polyneuropathy. Reported subjective mental workload for all tasks related to iBG-STAR was on average lower than 12 points, corresponding to the verbal code ‘nearly no effort needed’. A “Post Study System Usability Questionnaire”, evaluated the glucometer at an average value of 2.06 (SD = 1.02) on a 7-Likert-scale (1 = ‘I fully agree’ to 7 = ‘I completely disagree’) for usability. These results represent a positive user-experience. Patients with polyneuropathy may experience physical difficulties in completing the tasks, thereby affecting usability. Technologically savvy patients (n = 6) with a positive outlook on diabetes assessed the product as a suitable tool for themselves and would recommend to other diabetic patients. The main barrier to regular use was treating physicians’ inability to retrieve digitally recorded data. This barrier was due to a shortcoming in interoperability of mobile devices and medical information systems.

## Introduction

With more than 415 million diabetic patients globally, the demand for diabetes management is on the rise [1]. A cornerstone of therapeutic management of diabetes is blood glucose self-measurement [2–4]. This data is a crucial prerequisite for therapeutic decision making [5]. To date, patients have recorded blood glucose data with a pen and paper. Although this seems straightforward, documentation by hand requires substantial daily effort on the part of patients [6,7]. Recent advancements in mobile digital technology have facilitated the development of glucometer interfaces with mobile terminal to record, process and analyze blood glucose data, and present it to the user. [8–11]. This technology is very promising. Indeed, various studies have shown that mHealth solutions may increase treatment adherence and patient satisfaction if they are accepted by the user, including patients with no previous digital experience or with prior treatment regimens [12–14]. While iBG-STAR may contribute to diabetes management, user attitudes and expectations affect acceptance and usage of the device over time [15–17]. This explorative study examines the perception and usability of a glucometer connected to a smartphone with experienced diabetic patients. For this purpose, key aspects of modern glucometer performance were considered. Perception for implementation in routine care was explored by semi-structured interviews and assessed by the blood glucose meters’ usability, which was explored with simulated blood sugar measurements.

## Methods

### Ethics

The ethics committee of RWTH Aachen University was consulted and ethics approval issued (EK 028/15). In compliance to ethics committee recommendations, participants of this study simulated blood glucose measurement to minimize risk to participants. It was stipulated that no real lancet be used by the participant, and therefore no blood was drawn. For the purpose of simulating an authentic capillary blood collection, the lancet’s needle was exchanged for a blunt piece of metal dipped in red ink. The red colored injection point enabled participants to simulate blood collection using the test strip of the blood glucose meter. The device was set to demonstration mode so that no glucose reading was recorded. At no point during the study was it implied or encouraged that participants alter their standard therapy.

### Study design

In this explorative study, users performed several usability tasks as well as simulated blood glucose self-measurements with an Apple iPhone, while the think-aloud method was applied [18,19].

### Measuring technical affinity

Technological affinity was measured using a questionnaire addressing different topics (individual attitudes regarding comfort, efficacy, gender equality, control, dehumanization, interest and utility) [20]. There were 15 statements on different aspects of technology rated on a five-point Likert scale, ranging from ‘strongly agree’ to ‘disagree strongly’.

### Measuring health literacy

To gain further insight into the patients’ knowledge and ability to manage their disease, a health literacy questionnaire was applied [21,22]. This questionnaire assesses the patient’s access, understanding, evaluation, and application of health-related information. Additionally, the ability to assess information about disease prevention and to apply information to promote personal health was assessed with 16 questions rated on a four-point scale ranging from ‘very difficult’ to ‘very easy’.

### Measuring usability

The Post Study System Usability Questionnaires (PSSUQ) allowed for a quantitative assessment of usability [23]. The PSSUQ consisted of 11 questions rated by a five-point Likert scale, ranging from ‘strongly agree’ to ‘strongly disagree’.

### Measuring subjective mental workload

All usability tasks were evaluated by the Rating Scale of Mental Effort (RSME) to measure the subjective mental effort necessary for participants to accomplish the tasks [24,25]. The participants rated mental effort on a 150-point-scale. All participants were familiarized with the RSME scale using five appropriate daily life examples.

### Accessing users experiences and appreciated future functionality

Semi-structured interviews were constructed around central questions, addressing disease-specific contexts, data transmission, integration of the device into diabetological care, and ideas for extended functionality. Additionally, participants were asked how they experienced the simulated blood glucose measurement and what they liked or disliked about the device. Questions were designed according to norm DIN ISO 20282–1 [26].

### Tested system

The blood glucose meter”iBG-Star” manufactured by AgaMatrix Inc., Salem, USA in combination with iPhone 4S (16 GB) by Apple Inc., Cupertino, USA, was tested. Previous studies had shown the high accuracy of this device in measuring blood glucose [9]. Furthermore the related smartphone application was recently identified as one of the best for patient self-care [17]. For these reasons, the iBG-Star was chosen to model a new digital solution of treating diabetes and investigate barriers to integrating such devices into routine care of elderly diabetic patients.

### Data collection

Test sessions took place either at the participant’s home or at the institute. Each session began with a set of questionnaires. Patients were then introduced to the iBG-Star and performed usability tasks. They were asked to perform a simulated blood glucose self-measurement using the iBG-Star connected to an iPhone. The sessions concluded with structured interviews.

### Recruitment

Diabetic patients were recruited via local self-help groups by the Institute of Industrial Engineering and Ergonomics. All participants presented a minimum visual acuity of at least -.75 confirmed by a mobile visual acuity screening tool [27]. Participants were permitted to use standard vision aids. The principal investigator (PI) introduced the study, which was conducted independently and irrespective of medical treatment. Informed consent was a precondition for participation. Individuals already using a blood glucose meter connected to a smartphone physically or by certain data transfer protocols were excluded.

### Statistical analysis

Data were analyzed using the SPSS statistics software, version SPSS 22 (IBM).

## Results

### User statistics

n = 60 patients from two self-help groups in Aachen, Germany, were asked to participate into the study. n = 48 declined (6 stated they did not have access to a smartphone, 20 were not interested in taking part in ‘another’ study, 22 did not explain and 5 were already using a similar system). Of the 12 participating patients (median age of 66.5 years; range 30–78 years) 4 were male. n = 11/12 patients were suffering from diabetes (five from type I and six from type II) for more than 10 years. n = 4/12 patients reported diabetic polyneuropathy. n = 11/12 were on insulin-therapy (mean Hba1c 7.26%; SD = .598). All participants were familiar with daily glucose self-measurements (on average 4.25 measurements/ day; SD = 1.913). n = 8/12 documented with pen and paper, 4 stored data electronically on the blood glucose meter. N = 11 reported to present glucose data to their physician by paper diary at personal consultations. All participants finished at least secondary school, seven were retired, and all lived independently at home. n = 10/12 completed vocational education. All patients had prior experience with a smartphone or tablet, but no experience with the provided blood glucose meter. Every patient had a high level of technological affinity (mean 2.67; SD = 0.406) on a 4-point result scale ranging from ‘aversion’ to ‘affinity’). Measured health literacy showed high level of experience in all five dimensions, translating to capability in managing their disease. None of the participants had prior experience with a blood glucose meter that could be connected to a smartphone. However, all participants were highly familiar with smartphones and blood glucose self-management separately.

### Usability

According to the “Post Study System Usability Questionnaire” the investigated glucometer was evaluated at an average value of 2.06 (SD = 1.02) on a 7-Likert-scale (1 = ‘I fully agree’ to 7 = ‘I completely disagree’) for usability translating into a positive user-experience.

### Subjective mental workload

**Fig 1:**
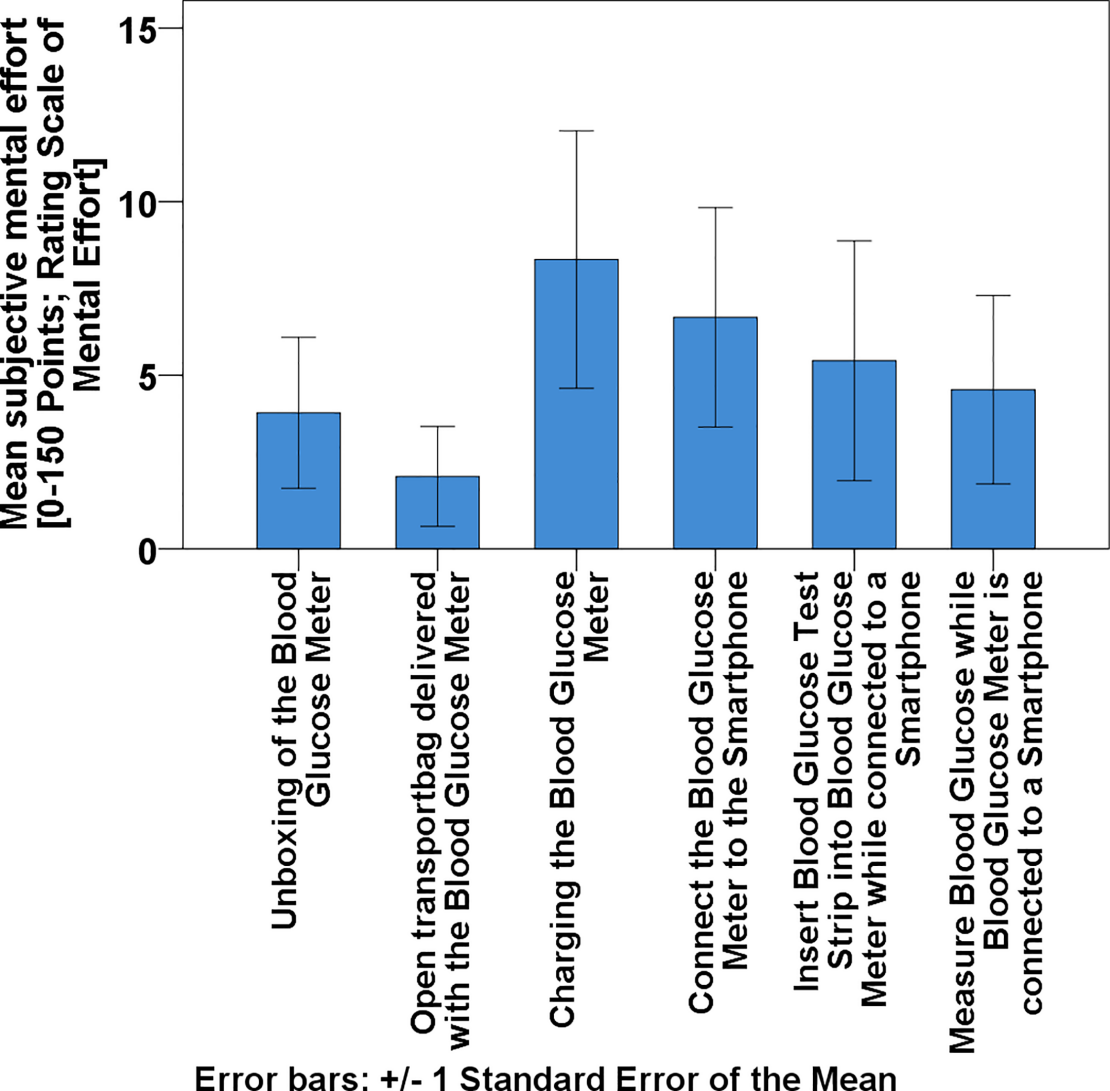
Mean subjective mental effort during initial contact and blood glucose measurement (Rating Scale of Mental Effort; 0–150 points scale).

The reported subjective mental workload for all tasks (i.e. charging, connecting to smartphone, inserting blood-glucose test strip, measuring blood-glucose) was on average lower than 12 points, corresponding to ‘nearly no effort needed’ (Fig 1).

### Interviews on user’s general experience

All patients stated that “iBG-Star” connected to a smartphone was as easy to use as a conventional glucometer. All patients reacted positively to the automated storage of measured blood glucose data in the smartphone app. Table 1 summarizes typical statements on device usability.

**Table 1:** Typical statements on usability of iBG-Star by patients.

Statement	Number of times mentioned in the interviews (N = 12)
“Automated documentation would be a great benefit for me.”	12
“The display of the glucometer itself is rather small and contrast is not sufficient.”	5
“With this glucometer I have to carry too many separate items with me (lancet for pricking the fingertip, testing strips, device, smartphone).”	5
“Automated data storage reduces my personal costs (in effort) for documentation.”	3

Qualitative analysis of interviews identified two subgroups among the participating patients. The first group (n = 6) had lower affinity for technology, were indifferent toward the iBG-Star, and preferred the “paper-system” for documentation. These users reported that they would recommend the new system to younger patients and patients without comorbidities. The second group (n = 6) expressed greater affinity for technology and a positive attitude toward their disease. This group found the product suitable both for themselves and for other diabetes patients. All patients with a history of polyneuropathy (PNP) (n = 4) were able to complete the simulated blood glucose self-measurement task. However, these patients required more effort in handling the lightning connector than those without PNP. Only n = 2/12 patients, both with a known history of PNP, assessed that the iBG-Star blood glucose meter may not be suitable during hypoglycemic events due to the size and loose fit of the testing strip.

### Interviews on users’ integration in routine care

Interviews revealed that all participants appreciated automated documentation of their blood glucose levels. Additionally, most (n = 10/12) participants expressed that they would share the data collected with their doctor or to an electronic patient record. This is a service not yet supported or offered by any doctor. One participant remarked in this context that “each manufacturer has its own standards on how to report or transfer the results”.

Half of all participants (n = 6/12) expressed that they would appreciate if the system was also compatible with non-Apple smartphones.

### Interviews on additional functionality

Semi-structured interviews revealed additional functions that participants would appreciate from a “digital” blood glucose meter. Table 2 summarizes typical statements that may be taken into account for future product development.

**Table 2:** Statements on possible extended functionality.

Statement	Number of times mentioned in the interviews (N = 12)
“I would appreciate being able to document physical activity or personal nutrition within the app.“	11
“I disapprove of direct transmission of data to my health insurance and the implementation of a bonus-malus program to foster therapy adherence based on this data.”	10
“I would appreciate if the system supported my physical activity or diet.”	8
“I would appreciate if the system (Smartphone + App) instructed me on how to use it (enough blood, testing strip okay, etc.).”	6

## Discussion

The present study is the first to systematically assess the usability of a self-measurement glucose-meter connectable to a smartphone, evaluate its potential contribution to diabetes management, and identify obstacles for implementation in regular care.

### Principal results

All participants expressed that the automated storage of blood glucose data is beneficial. Based on semi-structured interviews, the study identified barriers to the application of digital blood glucose meters independent from experiential usability. We demonstrated under laboratory conditions that iBG-Star is generally easy to use for elderly diabetic patients familiar with smartphones and blood glucose self-monitoring, yet in specific disease contexts (polyneuropathy, episodes of hypoglycemia) problems may arise that affect patient safety. The main obstacle to integrating blood glucose meters in routine care was found to be the lack of an easy-to-use option to transfer data to physician’s medical information systems. Additionally, qualitative interviews indicate that patients would like additional features and applications, i.e. recording of physical activity, nutrition or amount of administered insulin.

### Comparison with prior work

Only 6/ 60 of randomly approached diabetic patients already used an app-related blood glucose self-measurement system. This may be due to Germany lagging in general usage of health related mobile applications among elderly adults [28]. Our cohort was a subset of experienced diabetic patients who managed their disease relatively well according to the reported Hba1c and relied on pen and paper documentation. It has been shown that mHealth solutions can improve therapy adherence (i.e. drug-therapy, physical activity) and improve diabetic control [29]. All participants reacted positively to the automatic transfer of glucose data to mobile devices and possibly to an electronic health record, indicating the device’s potential to promote adherence and decrease the number of associated transmission errors [30–33]. Our data suggest that patients can generally handle this technology and appreciate its merit. The data further suggests that it may not be suitable for patients with polyneuropathy or for patients with limited visibility due to retinopathy. Our qualitative interviews showed that digital solutions for managing documentation are lacking. Although we recruited patients familiar with mobile technology, they continued recording data by pen and paper. Qualitative interviews confirmed the demand for holistic solutions for documenting diabetes-related data [17,34].

The results of our study reveal that one of the biggest obstacles from the patients' point of view is the interoperability of the device and medical information systems, and integrating the technology into standard care. Patients described their doctors as opponents of digitization, mainly since no standard of integration has been established. Doctors would be required to stock blood glucose meters from multiple manufacturers in order to support the integration of mobile technology into standard diabetic care. This problem of interoperability is well documented [17,35,36]. iBG-STAR resolves this challenge by automatically transferring data from the blood glucose monitor to the corresponding app. Despite this function, participants continued to report interoperability challenges with doctors unwilling or unable to receive digitally recorded data. This aspect of our study reveals a newly observed challenge, namely interoperability between patient and doctor.

An additional challenge revealed by our study is that while patients appreciated the ability to record health-related data electronically, they also expressed a desire to retain control of their medical information. This finding contributes to our comprehension of the limitations of digital data storage and transmission. Patients prefer managing their own data rather than having it automatically transferred to their insurers, where it might have an effect on the terms and benefits they receive. Aversion to the release of data was consistent even where patients may benefit from it, for example increased cost absorption. Future examinations should further investigate this tendency. This observation may be connected to the longstanding experience of participants in dealing with their illness.

Although digital care of diabetic patients has been studied and practiced for quite a while, implementation in daily care is lacking [37]. Patients continue to document their data by hand because electronic data transfer between physicians and their patients continues to pose a challenge. Our findings highlight the continued unresolved obstacles to the widespread use of mHealth, even among patients who recognize its usefulness.

### Limitations

A limitation of this study is the relatively small sample of participants, most of whom were women and, according to their Hba1c levels, adherent to diabetes medication. This limits the extension of the results to the general population. For example, the U.S. Food and Drug Association’s regulation “Applying Human Factors and Usability Engineering to Medical Devices” (FDA-2011-D-0469) accept a minimum 15 of participants in a usability assessment to approve new technology, making the sample examined in this study small [38]. Another limiting factor is that this sample was recruited locally from various self-help groups. Due to the structure of these groups, it can be assumed that these participants are very well informed about the self-management of their illness and accustomed to recording their blood glucose levels.

Future research could explore the usability of a device like iBG Star to promote diabetes management in young men at risk for lower adherence as well as among other groups identified as having insufficient diabetes self-management [39].

## Conclusion

In summary, the key to success of mHealth diabetes management technology is the development of a safe and low-threshold service that allows patients to control the transfer of data, and solves issues of interoperability between patients’ devices and doctors’ information systems. To achieve higher acceptance of mHealth tools in diabetes-care all stakeholders (industry, doctors and patients) should reach a consensus on data-transfer and security standards.
